# Emotion Beyond Bias: Affective and Cultural Scaffolding in Self-Regulation and Metacognition

**DOI:** 10.3390/jintelligence14100268

**Published:** 2026-10-17

**Authors:** Alice Conti, Alessandro Bortolotti, Alessandro Crociata, Maria Tartari, Pier Luigi Sacco

**Affiliations:** 1 Department of Neurosciences, Imaging, and Clinical Sciences, University of Chieti-Pescara, 66100 Chieti, Italy; alice.conti@phd.unich.it (A.C.); alessandro.bortolotti@unich.it (A.B.); 2 Department of Socio-Economic, Management, and Statistical Studies, University of Chieti-Pescara, 65127 Pescara, Italy; a.crociata@unich.it; 3 Institute of Heritage Science, National Research Council (ISPC-CNR), 80134 Naples, Italy; 4 metaLAB (at) Harvard, Cambridge, MA 02138, USA

**Keywords:** self-regulation, metacognition, emotion–cognition integration, achievement emotions, network neuroscience, predictive processing, interoception, cultural interventions, self-regulated learning

## Abstract

Intelligent performance depends on how cognitive ability is deployed, and its deployment is regulated by metacognitive and affective processes. This theoretical article concerns regulated deployment, on which the gap between maximal and typical performance depends, and leaves the structure of ability outside its scope. Influential decision-science frameworks treat emotion as a bias to be overridden by deliberation and design interventions accordingly; we argue that this view is difficult to reconcile with evidence that emotion and cognition arise from integrated, reciprocally connected networks, and we develop the view that affect functions as an organizing signal for monitoring and control. We derive a hypothesis that, to our knowledge, no study has yet tested directly: for challenges that require regulation to be regenerated over time, or the predictive models from which behavior flows to be reorganized, a culturally mediated route that engages affective, embodied, social, and aesthetic processes should yield change that lasts longer and transfers more widely than a content-matched route without them. We distinguish three classes of behavioral challenge by regulatory structure, locate self-regulated learning and metacognitive calibration within them, separate a testable claim about subjective regulatory effort from a conjecture about the formal control cost of brain-state transitions, and outline a test that could be preregistered.

## 1. Introduction: The Intelligent Mind as an Integrated Emotion–Cognition System

Intelligent performance in everyday life is rarely a matter of raw cognitive capacity alone. It emerges from the interplay between core abilities such as reasoning, memory, and attention and the self-regulatory processes that govern how those abilities are deployed: monitoring one’s own thinking, appraising and modulating emotional states, and adjusting strategy as circumstances change (Efklides, 2011; Zimmerman, 2002). Intelligence research draws the same divide when it distinguishes maximal performance, what people can do when they know that they are being evaluated, are motivated to do their best, and are assessed over a short period, from typical performance, what they do over the long stretches of ordinary activity (Sackett et al., 1988), and investment accounts attribute part of the difference to traits that govern how ability is invested over time (Goff & Ackerman, 1992; von Stumm & Ackerman, 2013). Contemporary cognitive neuroscience increasingly rejects a strict separation between cognition and emotion, instead portraying them as deeply integrated systems that jointly shape attention, working memory, executive control, and decision-making (Pessoa, 2008, 2013, 2022). Emotion runs through each of these processes: emotions influence which information gains access to limited cognitive resources, modulate the allocation of attentional effort, and bias the evaluation of alternative courses of action (Pessoa, 2008; Phelps, 2006), and cognitive strategies in turn regulate the emotions so generated (Gross, 2002; Ochsner & Gross, 2005). Furthermore, affective signals contribute to adaptive decision making under conditions of uncertainty by providing information relevant to the selection, maintenance, or revision of behavioral goals, thereby supporting rather than disrupting rational action (Bechara & Damasio, 2005; Damasio, 1994). An account of the intelligent mind in action must therefore assign emotion a constitutive role, as a component of intelligent behavior that helps individuals prioritize goals, allocate cognitive resources, and adapt to changing environmental demands (Ochsner & Gross, 2005; Pessoa, 2008).

Much of the decision sciences has taken the opposite path. An influential tradition treats emotion primarily as a source of bias and deliberative cognition as the corrective faculty that must override it (Kahneman, 2011). On this view, good performance consists in recruiting slow, analytic processing to discipline fast, affect-laden intuition, and the natural target of intervention is the choice environment that elicits biased responses (Thaler & Sunstein, 2008). The framework has been productive, and we do not dispute that some judgments are distorted in the ways it describes. Our concern is narrower: the architectural assumption that emotional and rational processing are separable systems standing in a corrective hierarchy is difficult to reconcile with what contemporary neuroscience has learned about how the brain implements affect and cognition.

The evidence that we review indicates that emotion and cognition emerge from integrated, reciprocally connected networks that resist any clean partition into competing subsystems (Barrett & Satpute, 2013; Pessoa, 2013, 2017). The extensive bidirectional connectivity between cortical and subcortical structures, the weak mapping between processing types and separable neural substrates, and the dynamic reconfiguration of large-scale networks according to task and state all favor co-regulation over hierarchy (Okon-Singer et al., 2015; Phelps, 2006). This picture carries an implication for self-regulation and metacognition that has so far been underdeveloped. If reflective control is realized through the same networks that carry affective signals, then interventions designed to bypass or suppress affective processing may forgo a principal lever on the system that they aim to change. We do not rest this claim on the architecture alone, since people can and do override affective impulses; it gains plausibility from behavioral evidence, drawn from separate literatures and never from head-to-head comparisons, that environmental and informational interventions tend to attenuate once withdrawn for behaviors requiring sustained regulation, whereas some affectively and socially engaging interventions show effects that persist.

This article develops that implication into a hypothesis and states how it could fail. The hypothesis is that emotionally engaging cultural and artistic practices support adaptive regulation by lowering the effort required to reach and hold reflective, goal-directed states, and that they achieve this by engaging the affective systems through which those states are accessed. It is comparative in a specific sense, since it concerns the relative efficiency, durability, and transfer of two routes to the same change, delivered with the same content and dose. We do not argue that affective engagement is sufficient for behavioral change, nor that cognitively oriented interventions never produce durable effects, since the evidence shows that some do (Gollwitzer & Sheeran, 2006; Hofmann et al., 2012). We hypothesize that for challenges requiring the continuous regeneration of self-regulation, or the reorganization of the beliefs and predictive models from which behavior flows, affective engagement is the more efficient, durable, and transferable route, and that culturally mediated practices are a particularly efficient way of providing it; the evidence that bears on this hypothesis is indirect, and the comparison that would test it has yet to be run.

### 1.1. Approach and Scope

This theoretical article develops its account from literatures selected for their bearing on the argument, without a standardized database search or formal screening protocol, so that its coverage is neither systematic nor exhaustive and it reports no pooled estimates. Two consequences follow for how the citations should be read: where a claim concerns the current state of an empirical question, we cite the most recent syntheses and report their design and magnitude, and where it concerns a definition, a theoretical position, or the origin of a construct, we cite the source in which it was established, as for the canonical statements of metacognitive monitoring (Flavell, 1979; Nelson & Narens, 1990), of achievement emotions (Pekrun, 2006), and of dual-process architecture (Kahneman, 2011).

The scope of the account follows from the distinction between maximal and typical performance drawn above. It concerns the regulated deployment of ability, the processes that decide whether, and how fully, the capacity measured under maximal conditions is brought to bear under typical ones, and it makes no claim about the structure or measurement of ability itself. The line between regulation and ability falls between deployment and capacity, since the control of attention is itself a component of fluid ability (Engle, 2002): the account concerns how much of an available capacity is deployed, for how long, and with what calibration, which can vary within persons while capacity remains constant. Regulation also shapes maximal performance when evaluation is threatening, as the effects of anxiety on attentional control show (Eysenck et al., 2007), although its influence is greatest under the extended and self-directed conditions of typical performance. Within that scope, it draws on network neuroscience, predictive and interoceptive inference, network control theory, and research on metacognition and self-regulated learning, together with three motivational literatures, self-determination theory, achievement goal theory, and models of self-regulated learning (Panadero, 2017; Ryan & Deci, 2017; Urdan & Kaplan, 2020), which Section 5.2 uses to treat affective engagement as a mechanism that regulates persistence, goal commitment, and the allocation of effort.

Several terms that overlap in ordinary usage are kept apart in what follows. Affective engagement is a property of an intervention’s design: an intervention engages affect when it is built to recruit emotional and interoceptive processes, independently of whether it later proves effective, so that the hypothesis cannot be rescued after the fact by redescribing a successful intervention as affective. Embodied, social, and aesthetic engagement are three modes of affective engagement, which recruit, respectively, bodily action and the interoceptive signals that accompany it, interpersonal coordination and the prediction of other people, and attention to the perceptual and symbolic form of an artifact. Cultural practices are socially transmitted and collectively elaborated forms of activity, such as ritual, narrative, music, dance, and theater, that carry shared symbolic content, and the arts are the subset of cultural practices organized around aesthetic form. A culturally mediated intervention delivers its content through such a practice, and so combines the three modes with features that belong to cultural forms, namely a structure refined over long periods, symbolic content, a voluntary and low-stakes frame, and availability at scale (Section 4.4); an affectively engaging intervention may recruit some or all of the modes without those features, as team sport or group psychotherapy do.

Because the argument joins findings of very different standing, we mark the standing of each link with five grades: direct evidence, where the link has been observed in the relevant population with designs that bear on it; indirect evidence, where it has been observed only in adjacent settings, outcomes, or designs; inference, where it follows from evidence together with theory without having been observed; hypothesis, where it is a prediction that existing methods can test; and conjecture, where it is a prediction that existing methods cannot yet test. 

### 1.2. The Propositions in Explicit Form

**Figure 1:**
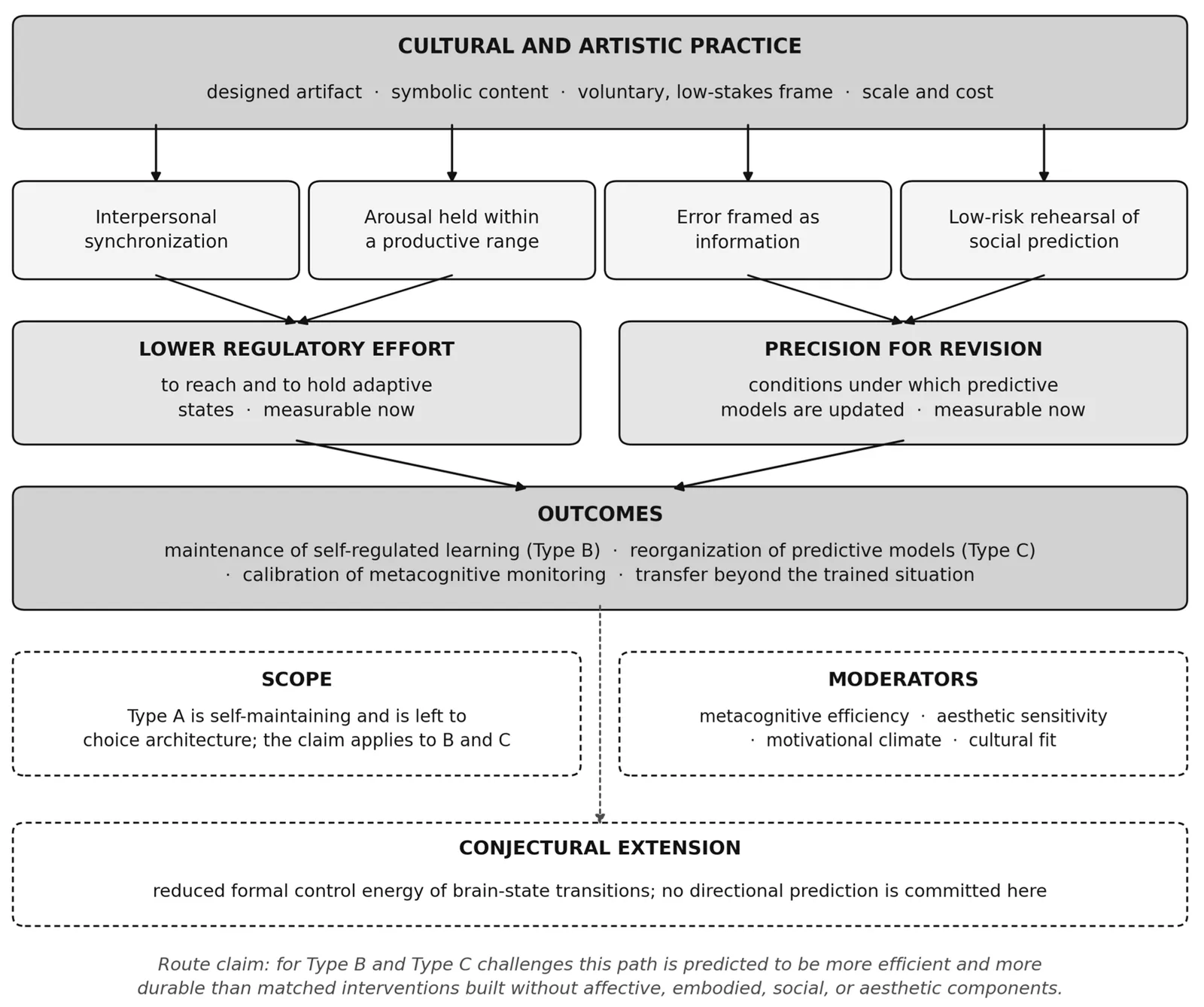
The proposed architecture. Cultural and artistic practice is characterized by four design features (Section 4.4) that engage four mechanisms (Section 4.2 and Section 4.3). These are proposed to act on two proximal quantities, the effort of occupying an adaptive regulatory state and the precision conditions under which predictive models are revised, both of which are measurable with existing methods. The links from design features to mechanisms rest on indirect evidence or on inference, and the links from mechanisms to the two proximal quantities, and from these to the outcomes, are hypotheses. The taxonomy of Section 3.3 fixes the range over which the route claim is asserted, and the extension to formal control energy is marked as conjectural.

Because the argument runs across several literatures, we state its content as four propositions before developing them. The article tests none of them, and they differ in standing: P1 summarizes convergent evidence, P2 is an inference from that evidence, and P3 and P4 are hypotheses, the second of which extends into a conjecture. Each is stated so that the observations that would count against it can be specified. Section 7.1 gives the disconfirmation condition for P3, and Figure 1 sets out the relations among them.

*P1, integration.* Emotion and cognition are realized in overlapping, reciprocally connected networks rather than in separable systems standing in a corrective hierarchy, so that what dual-process accounts describe as two kinds of process is better modeled as varying configurations of shared machinery (Section 2).

*P2, level.* Behavioral change that persists and transfers across contexts requires the reorganization of the beliefs and appraisals from which behavior flows, that is, of the parts of the generative model that represent the world and the self. Repetition within a stable context can maintain a behavior through habit (Wood & Rünger, 2016), which changes the responses cued by that context without reorganizing those beliefs, and interventions that act on the choice environment alone are not designed to reach them (Section 3 and Section 4).

*P3, route.* For challenges that require the continuous regeneration of regulation, or the reorganization of a generative model, which Section 3.3 defines as Type B and Type C, we hypothesize that a culturally mediated intervention produces model-level change that is obtained at lower cost, lasts longer, and transfers more widely to untrained contexts than an intervention that delivers the same content, with the same contact time, intensity, group format, and facilitator contact, without design features that recruit affective, embodied, social, and aesthetic processes. The route hypothesis, as we call it, is comparative in that it concerns the relative merits of two routes to the same change, and it makes no claim about whether either route works in absolute terms.

*P4, cost.* The mechanism proposed for P3 is a reduction in the effort required to reach and to hold adaptive regulatory states, which would free for the task some of the attentional resources that self-regulation otherwise consumes (Kanfer & Ackerman, 1989). This is a hypothesis at the level of subjective regulatory effort, which existing methods can measure, and a conjecture at the level of the formal control energy of brain-state transitions, which they cannot yet measure for the regulatory states at issue here (Section 4.1).

P3 implies two comparators: the content-matched intervention described in its statement, against which the hypothesis as a whole is tested, and an affectively engaging intervention that is not cultural, such as team sport or group psychotherapy, matched on arousal and social contact, against which the narrower claim that cultural forms add something of their own (Section 4.4) is tested. To our knowledge, neither comparison has yet been made directly, so the evidence reviewed in the following sections bears on P3 only indirectly.

The propositions carry with them two auxiliary commitments: response is expected to be heterogeneous rather than uniform, patterning with baseline metacognitive efficiency, aesthetic sensitivity, and the motivational climate in which an activity is delivered, so that a design which averages over these will both understate and misdescribe the effect; moreover, the constructs that the propositions employ are intended to be measurable with instruments already in use, which Section 7.2 sets out construct by construct.

The remainder of the paper is organized as follows. Section 2 sets out the positive neuroscientific case for emotion–cognition integration, including its predictive and interoceptive formulation. Section 3 turns that case into a critique of dual-process theory and choice-architecture interventions and introduces a taxonomy of behavioral challenges that specifies where each approach is expected to succeed. Section 4 develops the idea of culture as scaffolding through network control theory and predictive processing, distinguishes the two senses of cost on which the argument turns, and asks what is specific to cultural forms as against affectively engaging activity in general. Section 5 connects the framework to metacognition and self-regulated learning, the dimensions most directly relevant to intelligent performance, and reviews the intervention evidence bearing on it. Section 6 uses a case from the performance-art literature, and its replication record, to show why the comparison that P3 requires has to be run. Section 7 grades the evidence for each link of the account, sets out its scope conditions and the measurement of its constructs, outlines a test that could be preregistered, and discusses design implications and limitations, and Section 8 concludes.

## 2. Emotion and Cognition as Integrated Networks

### 2.1. Beyond Amygdala-Cortex Dualism

The intuition that emotion and reason occupy separate neural territories, with subcortical structures generating impulses that cortical structures restrain, remains embedded in dual-process thinking even where the psychological theory has moved on, although contemporary neurobiology does not support it. The amygdala, long cast as the brain’s emotion center, comprises multiple subnuclei with distinct connectivity profiles and behavioral roles that extend well beyond threat detection (Janak & Tye, 2015; LeDoux & Brown, 2017). The basolateral amygdala maintains bidirectional connections with much of the cortex, including regions associated with executive control and deliberation (Pessoa & Adolphs, 2010). Single-cell recordings identify distinct neuronal populations encoding positive and negative value, supporting rapid but graded evaluation of stimuli (Paton et al., 2006; Salzman & Fusi, 2010), and these signals are integrated with contextual information from the hippocampus and prefrontal cortex to guide behavior (Burgos-Robles et al., 2017; Kim et al., 2016; Tye et al., 2011).

The prefrontal cortex, for its part, does not simply inhibit the amygdala. Medial prefrontal projections can both amplify and attenuate amygdala responses depending on context (Quirk & Beer, 2006), while amygdala projections modulate attention, working memory, and decision-making so that affectively significant information receives prioritized processing (Pessoa, 2008; Phelps, 2006). The picture is one of continuous reciprocal exchange rather than one-directional control, and it is captured by the shift from a low road for rapid emotional responses to the recognition of many roads through which biological significance is evaluated (Pessoa & Adolphs, 2010). The amygdala’s role is better described as detecting biological relevance and resolving ambiguity than as generating fear as such; it is engaged by positive as well as negative events, and by uncertainty rather than threat alone (LeDoux & Brown, 2017; Salzman & Fusi, 2010). Even the paradigmatic emotional structure, therefore, does not behave as a dedicated affect module.

### 2.2. A Network Account of the Emotional Brain

These observations are systematized by network models of the emotional brain (Geng et al., 2024; Pessoa, 2017, 2022). Rather than localized emotion centers set against separate cognition regions, emotion and cognition emerge from dynamic interactions among large-scale networks. The salience network, anchored by the anterior insula and dorsal anterior cingulate, integrates affective and cognitive information to flag behaviorally relevant events; the default-mode network shows increased limbic coupling during emotional processing and reduced coupling during demanding cognitive control; and frontoparietal systems reconfigure their connectivity with both according to task demands, affective state, and individual differences (Bassett & Sporns, 2017; McMenamin et al., 2014). Consistent with this, the regions most often labeled as emotional, such as the anterior insula and dorsal anterior cingulate, are among the most domain-general in the brain, recruited across pain, effort, uncertainty, and cognitive control as readily as across affect (Pessoa, 2008). On this account, what dual-process theories describe as emotional versus rational processing corresponds less to two systems than to different configurations of shared neural machinery. The organizing question shifts accordingly, from which system holds control to which configuration the network currently occupies and what facilitates transitions between configurations. This reframing is the hinge of our argument.

### 2.3. Emotion in Predictive and Interoceptive Inference

A complementary account comes from predictive processing, which treats the brain as a hierarchical generative model that continuously anticipates its sensory input and revises its estimates when input departs from expectation (Clark, 2013; Friston, 2010; Parr et al., 2022; Rao & Ballard, 1999). Within this framework, emotion is best understood not as a discrete module but as arising from inference over the state of the body. The brain forms predictions about interoceptive signals, weighs them by their estimated reliability, and constructs affective states as its best explanation of their causes, categorized with the help of emotion concepts (Barrett, 2017; Barrett & Simmons, 2015; Seth, 2013; Seth & Friston, 2016). We stress the formulation because a looser version, in which emotions simply are prediction errors, misstates the theory: prediction errors are signals within the inferential process, whereas emotions are the inferred states that the process yields. Interoception, the sensing and integration of the body’s internal condition, supplies the substrate for this inference, with the anterior insula serving as a principal hub (Craig, 2009; Critchley & Garfinkel, 2017; Greenwood & Garfinkel, 2025).

A key feature of the account is the role of precision, the brain’s estimate of the reliability of a signal, which sets how strongly a given prediction error revises belief and action (Friston, 2010; Seth & Friston, 2016). Affective states are bound up with the regulation of precision, modulating how much weight is placed on interoceptive relative to exteroceptive evidence and thereby shaping attention and learning. The framework also situates emotion within allostasis, the anticipatory regulation of bodily resources in advance of need rather than in reaction to departures from a set point (Barrett & Simmons, 2015; Sterling, 2012). On this view, the brain’s core task is to keep the body viable by predicting its needs, and affect is the register in which the ease or difficulty of that predictive regulation is tracked. Emotion, so understood, is a continuous estimate of the organism’s predictive control of itself, which is one reason why it is not readily separable from the processes that support intelligent action.

The predictive and interoceptive view aligns closely with the network account (Pessoa, 2022). Both treat affect as an estimation process woven into perception, attention, and action rather than as an intrusion upon them (Greenwood & Garfinkel, 2025; Pessoa, 2022). We extend this convergence to intervention by proposing that durable change may require engaging the predictive models and bodily signals through which affective states are generated. Accordingly, we consider cultural and artistic practices as candidate means of reshaping predictive models and their precision.

### 2.4. From Integration to Intelligent Performance

Framing emotion and cognition as integrated has consequences for how we understand intelligent performance and its variation across individuals. If reasoning, memory, and attention are shaped by affective and interoceptive signals, their effective deployment also depends on emotion regulation and metacognitive calibration (Fleming, 2024; MacCann et al., 2020), and attentional control under stress provides a specific example of this dependence (Eysenck et al., 2007). Intelligence research offers two precedents for this view. The resource-allocation account of motivation and ability holds that self-regulatory activity draws on the same limited attentional resources that ability applies to the task, so that regulation competes with performance when the task is itself demanding, as in the early phases of skill acquisition (Kanfer & Ackerman, 1989). Individual differences in confidence, for their part, form a stable factor that is related to, and separable from, ability as measured by tests, which places the calibration of self-monitoring within the study of intelligence (Stankov, 2000; Stankov & Crawford, 1997). The importance of core abilities is undiminished by this view, which locates their expression within a regulatory context that determines whether and when they are brought to bear. We therefore propose that models of intelligent performance should incorporate the affective and metacognitive processes that govern the application of ability. The remainder of the paper treats that regulatory context as a primary target of both explanation and intervention.

## 3. A Neurocognitive Critique of Dual-Process and Choice-Architecture Approaches

### 3.1. Contemporary Dual-Process Formulations and What Survives Them

The canonical dual-process account positions System 1 as the source of systematic error and System 2 as the deliberative corrective (Kahneman, 2011). A serious critique must engage the most careful contemporary formulations rather than this popular version. Evans and Stanovich (2013) distinguish Type 1 processes, which are autonomous and do not draw on working memory, from Type 2 processes, which are working-memory dependent and support hypothetical thinking; they explicitly abandon the equation of Type 1 with emotion and Type 2 with reason, and describe the relation between the types as default-interventionist rather than as a simple contest. De Neys (2021) develops the account further, arguing that logical intuitions can themselves be Type 1, so that automaticity and error come apart. Taken to its conclusion, that argument narrows the functional work the two-type distinction can do, since the diagnostic property once assigned to Type 2, sensitivity to normative structure, is now available to Type 1 as well.

We regard these as genuine advances, and a full treatment of what follows for the architecture of decision is developed separately (Lokwani Di Matteo & Sacco, 2026). What matters here is a narrower point, and it is best made at the functional level on which the typology itself operates. The properties traditionally attributed to the two types, namely automaticity, efficiency, lack of awareness, and uncontrollability, dissociate rather than co-occur across tasks and measures (Melnikoff & Bargh, 2018). That is a finding about processing characteristics, not about anatomy, and it is therefore not answered by the standard and otherwise correct reply that a functional typology carries no commitment to separable neural substrates. Defenders of the typology reply, correctly for its careful formulations, that these properties are typical correlates and that the types are defined by autonomy and by dependence on working memory (Evans & Stanovich, 2013; Pennycook et al., 2018). The reply relocates the question without settling it, since dependence on working memory is, on our reading, a matter of degree across tasks and network configurations, and the logical intuitions documented by De Neys (2021) show that sensitivity to normative structure does not track it. A framework in which processing features vary continuously and quasi-independently across distributed network states predicts the observed dissociations and requires no cut on that continuum, whereas a two-kind typology must place a boundary for which the evidence supplies no principled location. The neuroscience of Section 2 then offers support rather than decisive arguments: it offers a mechanism for why the features should be expected to dissociate, since they are properties of configurations of shared machinery rather than signatures of distinct systems.

A second commitment is more resistant. The residual association of bias with Type 1 preserves an evaluative asymmetry that obscures how deliberation is itself systematically biased, a point conceded even by sympathetic authors under the heading of dysrationalia (Stanovich, 2018) and reinforced by evidence that thinking biases are only weakly related to cognitive ability (Stanovich & West, 2008). If reflective processing has its own characteristic failure modes, and if greater cognitive capacity does not reliably protect against them, then the corrective architecture is doing less explanatory work than its role in the framework suggests. Our aim is not to deny that processing varies in speed, automaticity, and working-memory demand, but to question whether those dimensions carve cognition into two kinds, or whether they are better modeled as varying properties of distributed network states.

### 3.2. From Bias to Evolutionary Mismatch and Positive Affect

A second line of criticism concerns the interpretation of bias itself. Many phenomena catalogued as failures of reasoning can be read as instances of evolutionary mismatch, in which heuristics fitted to the environments where they took shape misfire in environments that differ from them (Gigerenzer & Gaissmaier, 2011; Sacco, 2025). The rationales proposed for particular biases, such as the reduction of costly conflict by status-quo tendencies in stable groups, the reasonableness of steep discounting when future resources were uncertain, and the tracking of frequency by availability in information-sparse settings, are inferences about the fit between heuristic and environment, which the mismatch reading makes plausible without establishing them. A related argument locates the rationale of several canonical biases in the metabolic economics of neural computation, on which apparently suboptimal inference is efficient once the energetic price of exact inference is counted (Sacco, 2026b). On either reading, the appropriate corrective is less the sporadic activation of deliberation than the recalibration of heuristics for contemporary contexts, and culture, which evolves far faster than biology, is a plausible vehicle for that recalibration because it engages emotional and social cognition rather than bypassing them.

Positive affect reinforces the point. The broaden-and-build account holds that positive emotions widen the scope of attention and cognition and accumulate durable psychological resources (Fredrickson, 2001; Fredrickson & Branigan, 2005). Proposed neurobiological explanations have linked these effects to dopaminergic modulation of cognitive flexibility (Ashby et al., 1999). Early studies linked positive affect to flexible problem-solving and to greater integration of information in judgment (Estrada et al., 1997; Isen, 1999). A systematic review and meta-analysis of experimental inductions of positive affect, pooling nineteen effect sizes, found no overall benefit for inhibition, working memory, or cognitive flexibility (Lautenbach, 2024), although a subsequent pair of small experiments by the same author recovered an effect on cognitive flexibility specifically (Lautenbach, 2025), which suggests an effect narrower and more condition-dependent than the broaden-and-build account anticipates. Self-transcendent states illustrate the integrative character of affect with particular clarity: awe combines diminished self-focus with heightened accommodation of novel information (Monroy & Keltner, 2023; Piff et al., 2015; Stellar et al., 2017). Such states sit uneasily on a single automatic-to-deliberative axis, which is what the network account leads one to expect.

### 3.3. What Choice Architecture Can and Cannot Do

These considerations bear directly on choice architecture. Nudging restructures the environment so that preferred options are easier to select at the moment of decision, without foreclosing alternatives (Thaler & Sunstein, 2008). Its standard rationale is itself dual-process, on which conventional interventions address deliberative processing through information and incentives while nudges act on automatic processing by reshaping the environment in which choices are made (Kahneman, 2011; Thaler & Sunstein, 2008). That rationale has fared poorly under scrutiny, since susceptibility to nudges turns out not to depend on whether a decision is made in an automatic or a deliberative mode, nor on whether the nudge is disclosed to the person being nudged, while it does depend on whether the nudged option is one that the person already wanted (de Ridder et al., 2022). The instrument survives the architecture that was supposed to explain it, which is what the argument of Section 3.1 would lead one to expect.

The aggregate magnitude of the effect has been disputed as sharply as its mechanism, with an influential meta-analysis reporting a small to medium pooled effect (*d* = 0.43; Mertens et al., 2022) and subsequent re-analyses arguing that this estimate is inflated by publication bias to the point of leaving little residual effect (Maier et al., 2022; Szaszi et al., 2022). That dispute has now been addressed at a higher level of aggregation. A second-order meta-analysis synthesizing thirteen articles that report fourteen meta-analyses, covering 1,638 primary studies and approximately 30 million participants, recovers a small positive pooled effect (*d* = 0.27, 95% CI [0.16, 0.38]) that survives Bayesian model averaging across publication-bias models (*d* = 0.29) and falls to approximately zero once small-study effects are corrected (*d* = 0.004), against a background of extreme heterogeneity (*I*^2^ = 99.89%) and generally low methodological quality among the underlying reviews, of which only three were preregistered (Hu et al., 2025). The authors note that such corrections can overcorrect and that their synthesis inherits the weaknesses of its inputs, and they conclude that nudges work modestly and unevenly and complement structural and incentive-based instruments.

We draw two consequences from this. First, if the aggregate effect is smaller and more fragile than the field had believed, that fragility extends to the Type A challenges (see Table 1) where we expect choice architecture to be the efficient instrument, so the first row of the taxonomy below rests on ground that is itself under revision. The concern is tempered by the fact that decision-structure nudges, the category containing defaults, carry the largest point estimate (*d* = 0.40, against 0.23 for decision-information nudges), although the difference is not statistically reliable in a moderator analysis conducted at the level of reviews (Hu et al., 2025).

The second consequence concerns where nudges do and do not work, and the pattern anticipates the distinction that we are about to draw. Pooled effects are positive in the environmental (*d* = 0.45) and food (*d* = 0.33) domains (Cadario & Chandon, 2020; Hu et al., 2025), and negligible in health (*d* = 0.17, with a confidence interval spanning zero). Environmental and food outcomes are dominated by selections made once at a point of decision, whereas the behaviors that dominate the health domain, namely adherence, physical activity, and dietary maintenance, require the behavior to be produced again on each occasion. The authors of the synthesis reach a compatible reading, suggesting that entrenched health behaviors may require instruments stronger than nudges. None of the moderators tested, domain and setting alike, reached significance in their meta-regression, so the pattern is one to explain rather than to build on. Effects also shrink at scale: trials run by two government nudge units show effects about one sixth the size of those reported in academic journals, 1.4 against 8.7 percentage points (DellaVigna & Linos, 2022).

Alongside magnitude sits the question of persistence, where the evidence is mixed in an instructive way: the effects of repeated behavioral interventions decay slowly once treatment stops, by 10 to 20 percent a year for home energy reports, leaving a residue consistent with habit formation (Allcott & Rogers, 2014), and one-off structural changes such as defaults require no such repetition to persist. Taken together with the domain pattern, this suggests that nudges shift choices at the point of decision, that their magnitude and durability vary with what the behavior demands of the person afterwards, and that the variation is systematic enough to be worth a classification.

All of this is intelligible in the terms of Section 2. A nudge alters the external conditions of choice; it does not, in itself, alter the internal models and affective signals that make a regulated state easy or hard to occupy. Where the desired behavior persists without ongoing regulation, this is sufficient. Where the behavior must be regenerated repeatedly against competing demands, we infer that environmental support substitutes for regulation without building it, so that behavior tends to revert once the support is gone, except for whatever residue repetition has left in the form of habit. A useful way to make the boundary precise is to distinguish three classes of behavioral challenge by their regulatory structure, defined before and independently of any intervention. Table 1 sets out the classes, their exemplars in the behavior-change literature and in the study of learning, and the intervention fit that we predict for each.

Because the classes are defined by regulatory structure, independently of which intervention works on them, the mapping to intervention in the final column is an empirical prediction, and it can be wrong. One qualification is immediate: some Type B behaviors can be durably solved by environmental engineering that removes the regulatory demand without building regulation, as long-acting medication formulations or unit-dose packaging do for adherence, so the prediction is properly that choice architecture fails for Type B challenges that remain regulatory.

The classes correspond to distinctions that the behavior-change literature already draws, and the correspondence shows what the taxonomy adds. Type A matches the problem of initiation, which a single well-placed decision can solve, and Type B matches the problem of maintenance, for which a systematic review of behavior theories identifies five recurring themes, namely maintenance motives, self-regulation, psychological and physical resources, habit, and environmental and social influences (Kwasnicka et al., 2016; Rothman, 2000). Two of these themes bear directly on the boundaries between the classes. Habit maintains a behavior through context-cued automaticity after intentions have ceased to do so (Wood & Rünger, 2016), and in the terms of active inference it corresponds to learned priors over policies, a form of model change confined to the contexts in which the policy was repeated, which leaves untouched the beliefs about the world and the self that govern behavior across contexts (Friston et al., 2016; Parr et al., 2022). This is why repeated environmental interventions can leave a residue after they are withdrawn (Allcott & Rogers, 2014), and why such a residue is vulnerable to changes in context that remove the cues on which it depends (Verplanken & Wood, 2006; Wood et al., 2005). Internalization, which self-determination theory describes as the movement of regulation from external contingency toward integration with the self (Ryan & Deci, 2017), maintains behavior through endorsement, and it generalizes across contexts to the extent that it reaches the beliefs and values that govern a family of behaviors, which is the target that defines Type C. Nudges act on the choice environment and fit Type A, whereas boosts aim at competences and therefore at Types B and C (Hertwig & Grüne-Yanoff, 2017). What the taxonomy adds is a classification of the challenge, made before and independently of any intervention, which ties each class to the cost of the state transitions that it demands and extends the classification to learning and metacognition. The correspondence with habit also fixes the outcome on which the route claim turns: because habit can make a Type B behavior self-maintaining within a stable context, persistence alone cannot discriminate between routes, and the discriminating outcome is persistence together with transfer to contexts in which the behavior was not practiced.

Because the taxonomy is meant to be applied, we state the classification rule explicitly, as three questions that settle most cases. The first asks whether the behavior, once selected or performed, persists without further regulatory input from the person, and where it does the challenge is of Type A. Where it does not, the second and third questions are both asked. The second asks whether the same behavior must be produced again on each occasion against competing demands, and a positive answer makes the challenge Type B; the third asks whether the intended target is a belief, appraisal, or predictive model that governs a family of behaviors, and a positive answer makes it Type C, so that a challenge answering both positively is mixed. Three conventions handle what the questions leave open. Classification is relative to a declared outcome and time horizon, so that one policy presents a Type A challenge for enrollment and a Type B challenge for adequacy over a career, and the analyst has to say which is at issue. A mixed case is assigned to the component that dominates variance in the declared outcome, with the subordinate component recorded, since it is what a secondary analysis would test. And a challenge can be moved from one class to another without building regulation, by engineering, as long-acting formulations do for adherence, or by habit formation within a stable context, so the classification describes the challenge as currently posed and not a fixed property of the behavior. Table 2 applies the rule to cases drawn from both literatures, including mixed cases in which the declared outcome decides the class.

The prediction is also, in the form in which we state it, untested. Existing syntheses code nudges by technique and by behavioral domain and report no reliable moderation by either (Hu et al., 2025), which might be read as evidence that no classification of this kind will pay off. Regulatory structure cuts across both such codings, since a single technique can be aimed at challenges of any of the three classes and a single domain contains all three, so a null result for technique and domain leaves a classification that nobody has coded untouched. What follows is a concrete, inexpensive proposal: recoding the trials already assembled in these meta-analyses by the regulatory structure of the behavior that each one targeted, and testing whether that variable moderates effect size and persistence where technique and domain do not, would make the taxonomy testable on evidence that has already been collected.

The fourth column of Table 1 is where the framework becomes testable for the domain of intelligent performance. Acquiring and sustaining self-regulated learning is a Type B challenge, since strategy monitoring and effort allocation must be regenerated on every occasion of study rather than settled once. Changing what a learner believes about the nature of their own ability is a Type C challenge, since the target is a model that governs behavior across many situations, and recalibrating the felt cues by which a learner judges whether something has been understood becomes one when the declared outcome is resolution on untrained tasks (Table 2). The best-studied intervention on beliefs about one’s own ability is informational and brief. Growth-mindset interventions delivered as short online modules raised grades among lower-achieving students in a national experiment, with effects that depended on the norms of the school (Yeager et al., 2019), and meta-analyses report small average effects, larger among students expected to benefit most, such as those at academic risk (Burnette et al., 2023; Sisk et al., 2018), which become negligible in one synthesis once study quality and bias are taken into account (Macnamara & Burgoyne, 2023). Such evidence cannot discriminate between P3 and its rivals, since small and context-dependent effects are expected on several accounts, and no study has compared such interventions with an affectively and culturally delivered counterpart; the paradigm, however, offers a ready test of P3 in the domain of intelligence, to which Section 7.3 returns. Boosts, which aim to build competencies rather than exploit defaults, extend the reach of choice-based approaches toward the capacity-building that we advocate. Insofar as they remain narrowly cognitive, we expect them to encounter the same limitation for Type B and Type C challenges, where the target states are affectively constituted, though this is a comparative prediction rather than a stipulation, and we return in Section 7 to the interventions that press hardest against it. The remainder of the paper concentrates on Type B and, especially, Type C.

## 4. Culture as Scaffolding: Network Control, Effort, and Error Framing

This section addresses one question: how emotionally engaging cultural practices might reduce the cost of occupying adaptive regulatory states and support the revision of the predictive models from which behavior flows. The question decomposes into four steps that carry different evidential weight and are treated in separate subsections for that reason, since a merged treatment would obscure the difference. Section 4.1 fixes the two senses of cost on which everything downstream depends, and separates the claim that current methods can test from the conjecture that they cannot. Section 4.2 takes the first half of the question, the cost of reaching and holding a state. Section 4.3 takes the second half, the conditions under which prediction error is used for revision rather than defended against. Section 4.4 then asks what, if anything, is specific to cultural forms, which is the objection that the two preceding subsections invite.

### 4.1. Brain State Transitions and the Two Senses of Cost

If adaptive behavior depends on occupying an appropriate network configuration, the central practical question is what it costs to reach that configuration and to remain in it. Network control theory offers a formal treatment. Modeling neural dynamics as a linearized dynamical system evolving on the structural connectome, it quantifies the control input required to drive the brain from one activity state to another and shows that this cost depends systematically on network topology (Betzel et al., 2016; Gu et al., 2015; Lynn & Bassett, 2019; Parkes et al., 2024). Densely connected hubs in default-mode regions such as the precuneus and posterior cingulate exhibit high average controllability, from which many nearby states are reachable at low cost, whereas transitions to more distant configurations are often more costly, particularly when the target lies far from the current state (Betzel et al., 2016; Gu et al., 2015). The framework distinguishes several notions that are useful here. Average controllability indexes the capacity to move the system into many nearby states with modest input; modal controllability indexes the capacity to drive the system into distant, difficult-to-reach states, and is characteristic of more weakly connected regions (Gu et al., 2015). Adaptive cognition requires both, namely the ability to settle efficiently into common configurations and the ability, at times, to reach states far from the current one. Developmental analyses indicate that the energy required to support executive states declines across adolescence as structural connectivity matures (Cui et al., 2020). What the theory does not yet tell us is how experience of particular kinds, including cultural and affective experience, reshapes these quantities.

Because the construct is easily over-read, we separate two senses of cost and attach our claims to each explicitly. The first is regulatory effort in the ordinary, phenomenological sense: how difficult it feels to enter and maintain a state in which demanding work can be undertaken, how much of the available regulatory capacity that maintenance consumes, and how quickly it decays. This quantity is measurable now, through subjective effort and workload ratings, persistence under load, pupillometric and cardiac indices of exerted control, and the temporal profile of performance across sustained tasks. The second is control energy in the formal sense, the magnitude of the input required to steer the modeled system from one state to another. These are distinct quantities that are plausibly but not demonstrably related, and neither is identical to metabolic expenditure, although the metabolic price of precision-weighted inference is one candidate bridge between them (Sacco, 2026b). P3 and P4 are formulated at the level of subjective regulatory effort, which existing methods can test, and their extension to formal control energy is a conjecture. Except where we invoke the formal metric explicitly, cost and effort are used in the ordinary sense.

**Figure 2:**
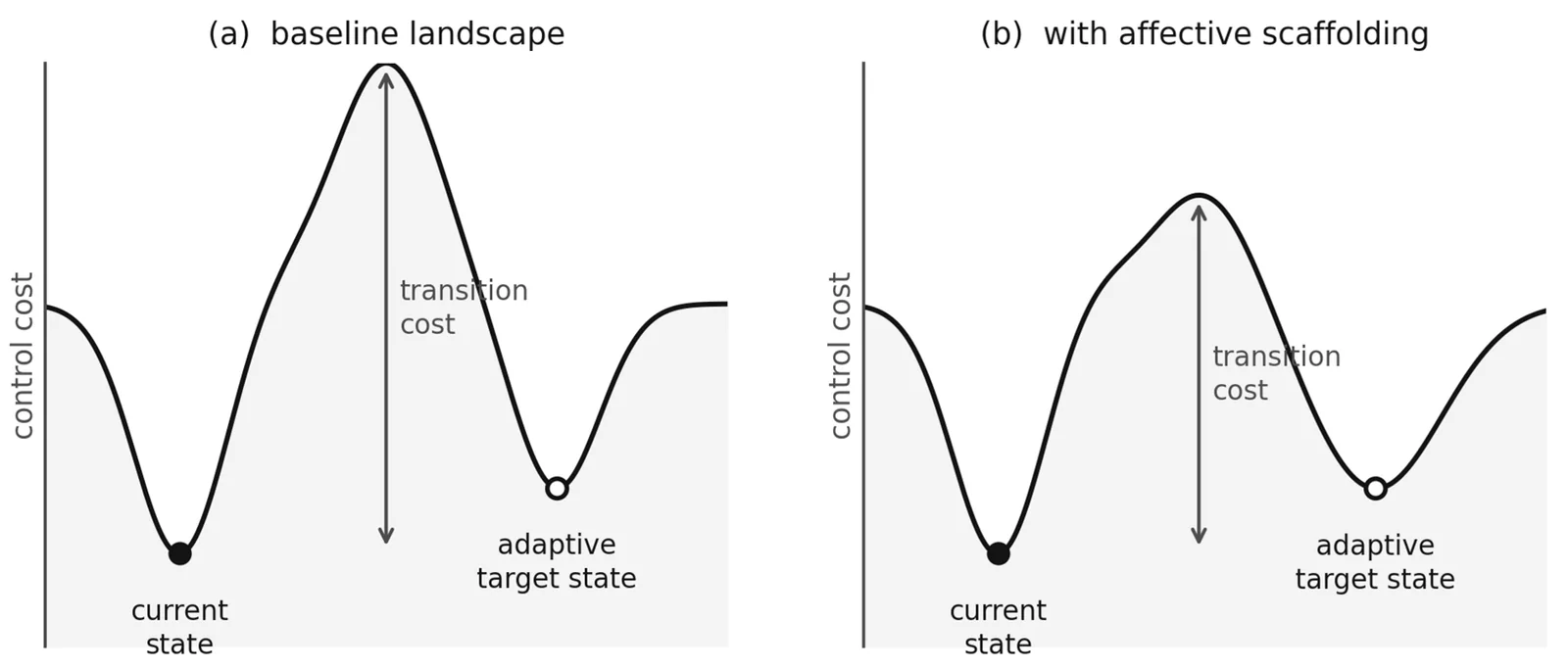
The role of affective and cultural scaffolding, illustrated. Panel (**a**) shows a schematic landscape in which the transition from the current state to an adaptive target state passes a high ridge; panel (**b**) shows the same transition under affective and cultural scaffolding, with a lower ridge and a broader target basin. The figure illustrates the claim developed in Section 4.1 and Section 4.2 and reports no simulated or empirical data. That claim is a hypothesis about subjective regulatory effort, and its extension to formal control energy remains a conjecture (Table 3).

The distinction also disposes of an objection that would otherwise be serious. A robust finding in the science of learning is that certain desirable difficulties, such as spacing, retrieval practice, and interleaving, improve durable learning precisely by making study effortful, and that the subjective ease that learners take as a sign of mastery is often an illusion (Bjork & Bjork, 2011; Dunlosky et al., 2013). A framework built on lowering effort might seem to contradict this. It does not, once the effort that desirable difficulties add, which is cognitive effort at encoding and retrieval, is distinguished from the regulatory effort of reaching and staying in a state where difficult cognitive work can be undertaken at all and of tolerating the errors and uncertainty that learning entails. We propose to lower the second so that the first can be sustained. Affective and cultural scaffolding should make hard learning bearable rather than easy (Figure 2), and it inherits the caution that fluency-based comfort must not be mistaken for competence.

Three further limits belong to the formal apparatus. The target states in this work are defined as patterns of neural activation rather than by psychological labels, so attaching specific psychological content to a control-theoretic target, such as prosocial orientation, requires an operationalization that has largely still to be carried out. The developmental evidence that transition energy is lower later in adolescence is cross-sectional and manipulates no experience (Cui et al., 2020); it is consistent with the landscape changing over development, but does not establish that experience shapes it, or that intervention can modify it, and we treat experience-dependent modifiability as an open possibility. Finally, the framework assumes linearized, noise-free dynamics whose adequacy for neural activity is debated, and its controllability metrics are strongly shaped by nodal connection strength; we therefore use it as a source of precise concepts and testable predictions rather than as a settled account (Lynn & Bassett, 2019; Parkes et al., 2024).

### 4.2. How Cultural Practices May Lower the Cost of Adaptive States

Within these constraints, we hypothesize that emotionally engaging cultural practices facilitate transitions to adaptive states and reduce the subjective effort required to maintain them, and two timescales should be kept apart in assessing the hypothesis.

On the acute timescale, several lines of evidence are consistent with the inference that cultural engagement reconfigures distributed networks toward integrations that would otherwise be costly to reach. Hyperscanning studies show inter-brain synchronization during collective music-making that extends beyond motor coordination (Müller et al., 2013), which is consistent with participants converging on shared predictive states, although such synchrony can also reflect common sensory and motor input, so the functional reading remains tentative. Neuroimaging of aesthetic experience shows strong engagement of reward circuitry, including dopamine release in the nucleus accumbens measured with positron emission tomography during peak emotional responses to self-selected music (Salimpoor et al., 2011), alongside recruitment of default-mode regions during intense aesthetic experience (Vessel et al., 2012), consistent with a temporary integration of reward and self-referential processing. Candidate neurochemical substrates include endogenous opioid release during synchronized movement (Tarr et al., 2014) and context-dependent oxytocinergic modulation of social cognition (Shamay-Tsoory & Abu-Akel, 2016). These lines of evidence are convergent but indirect: none measures the controllability of the connectome, and the inference from acute connectivity change to reduced control cost remains to be tested directly.

On the longer timescale, sustained cultural practice is associated with structural change. Music training, dance, and contemplative practice are linked to differences in white-matter integrity and gray-matter density (Karpati et al., 2015; Schlaug, 2015; Tang et al., 2015). Because the cost of state transitions depends on this structural architecture, sustained practice may alter the energy landscape for future transitions. The inference is plausible but not demonstrated, and to our knowledge no study has yet linked a cultural intervention to a change in controllability metrics.

Beyond synchronization and plasticity, we infer that cultural forms modulate arousal within bounds that favor flexible transition. The inference rests on the general relation between arousal and performance more than on studies of the arts. The relation between arousal and performance is characteristically curvilinear, with intermediate arousal supporting performance and extremes impairing it, a regularity associated with the Yerkes-Dodson tradition and refined by later work that emphasizes its task dependence (Arent & Landers, 2003; Hanoch & Vitouch, 2004). Rhythmic entrainment, narrative tension and resolution, and calibrated novelty are among the devices through which artistic practices can hold participants within a productive arousal range, while ritual and narrative structures supply external scaffolding for transitions that would otherwise be difficult to complete. Aesthetic settings designed with this in mind have been reported to reduce anxiety and stress in museum visitors (Banzi et al., 2026), and the aesthetic organization of complexity itself has been proposed as a mechanism by which such settings hold attention without overwhelming it (Bortolotti et al., 2025).

### 4.3. Predictive Processing and Error Framing

Predictive processing provides a second mechanism. If learning is driven by prediction error, the conditions under which error is encountered matter greatly. Ordinary attempts at behavior change generate prediction errors that are often experienced as threat: the discomfort of disrupted routine and the uncertainty of unfamiliar action recruit defensive responses that narrow exploration. How error is framed has measurable consequences for what is learned from it: training and work settings that treat error as information to be exploited rather than as failure to be avoided yield better adaptive transfer than settings that treat it as something to be prevented (Frese & Zapf, 1994; Keith & Frese, 2008; Steele-Johnson & Kalinoski, 2014). We infer that artistic and cultural settings characteristically instantiate the informative framing, although no study has compared them with error-management training on this dimension: improvisation treats the unexpected as material to build on, and ensemble music generates controlled violations of expectation that carry affective weight without the stakes that would trigger defense. Dopaminergic signaling offers a candidate substrate, since the phasic reward prediction-error response begins with a brief, unselective component that detects a wide range of stimuli before evolving into a component that reflects reward value, so that the response trades accuracy for speed at the moment of detection (Schultz, 2016). We note, however, that reward prediction error in this sense is related to but distinct from the interoceptive prediction errors from which affective states are inferred (Section 2.3), and the two should not be conflated.

**Figure 3:**
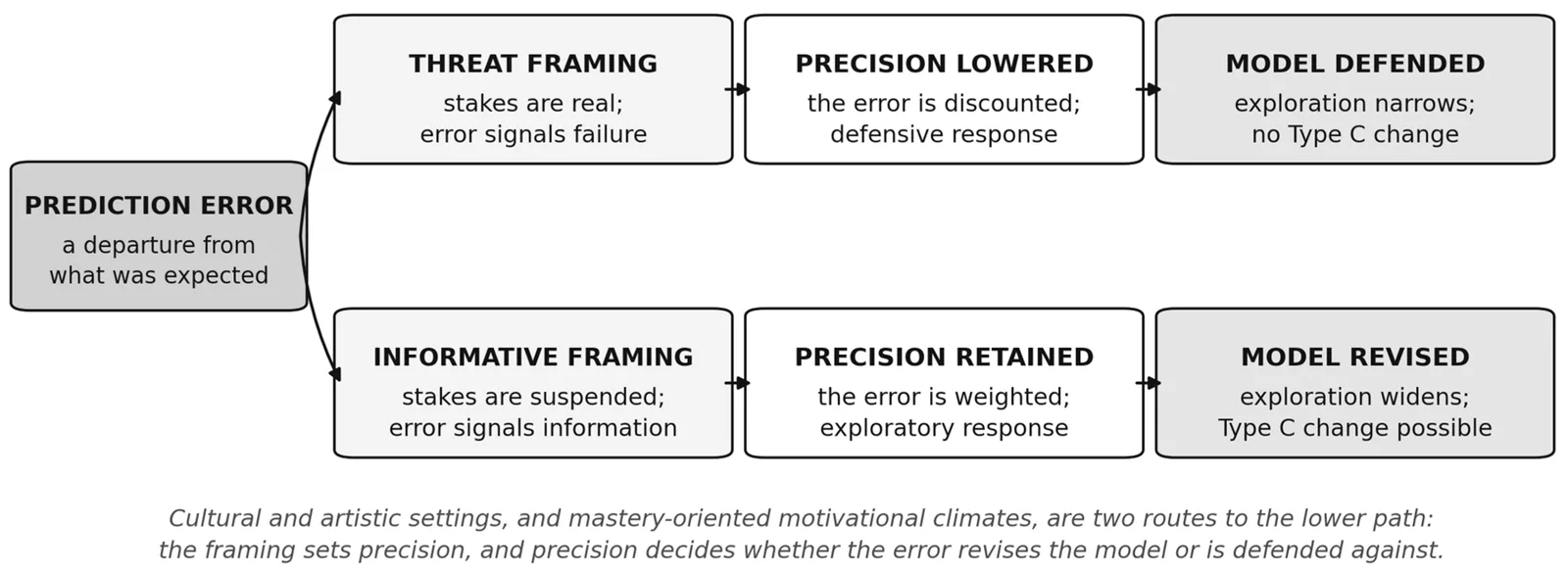
Error framing and precision. On the proposed account, the same prediction error follows one of two paths according to the framing under which it is encountered. Threat framing lowers the precision assigned to the error and recruits a defensive response, so the model is preserved and exploration narrows; informative framing preserves precision and permits revision. Cultural and artistic settings, and the mastery-oriented motivational climates discussed in Section 5.2, are proposed as two routes to the second path.

Framed in terms of active inference, error framing points to a further function (Figure 3). Agents do not only minimize prediction error against fixed expectations; they also act to gather information that will improve their models, a drive often described in terms of epistemic value or the resolution of uncertainty (Friston et al., 2015; Seth & Friston, 2016). Cultural and artistic settings may be well suited to supporting this exploratory drive, because they present novelty and ambiguity within a frame that signals safety, so that uncertainty which would otherwise prompt avoidance becomes an occasion for exploration. Play, narrative, and aesthetic experience can in this sense be understood as culturally elaborated means of safe epistemic foraging, in which predictive models are stretched and revised without the costs that the same revisions would carry in unmediated experience (Bortolotti et al., 2024).

The same framework suggests why cultural practice could rehearse social prediction at low risk. Narratives, songs, and rituals present social scenarios in which models of cooperation, trust, and repair are refined without real-world consequences, a mechanism that has been proposed as a route to emotional regulation in domains where direct experience is costly or dangerous to acquire (Tartari et al., 2026). This is especially relevant to intergroup behavior, where the relevant predictive models are otherwise updated only through infrequent and demanding direct contact, and it connects the mechanistic account to the case examined in Section 6.

### 4.4. What Is Specific to Cultural Forms

The mechanisms surveyed so far, namely synchronization, arousal modulation, error reframing, and low-risk rehearsal of social prediction, are not the exclusive property of the arts. Team sport, group psychotherapy, collective worship, and military drill recruit several of them, and any adequate theoretical account of culturally mediated change must specify what cultural and artistic forms add. Four candidate features may distinguish them, and each is in principle separable and testable.

The first is that cultural forms are designed artifacts whose structure has been selected over long horizons for the reliable induction of particular inferential and affective states. Traditions, norms, and customs stabilize social prediction by distributing shared expectations, whereas arts and media supply simulated experiences that expose the predictive machinery to alternative social realities and inject controlled variability into it; the two operate as complementary adaptations of the same predictive apparatus (Bortolotti et al., 2024). If this is correct, cultural repertoires arrive pre-optimized in a way that ad hoc affective engagement does not. The hypothesis concerns efficiency and can be tested by comparing culturally elaborated formats against matched but unrefined ones.

The second is symbolic content. Arousal modulation and synchrony operate on state; narrative, image, and metaphor operate on the model, because they present specific propositional and relational content about persons, causes, and possibilities (Bortolotti et al., 2024; Tartari et al., 2026). Shared narratives, rituals, and symbolic representations may in this way function as socially distributed regulatory resources that reduce reliance on individual cognitive control, which connects the account to sociocultural theories of learning and of collective sense-making, and to integrative evidence associating arts participation with several dimensions of social cohesion, where the causal pathways remain insufficiently established (Sonke et al., 2025). A framework whose principal target is the generative model rather than the individual act therefore has a particular reason to prefer forms that carry semantic structure, which is what distinguishes Type C reach from the Type B benefits that many affectively arousing activities can deliver.

The third is that some forms of aesthetic engagement are voluntary and non-instrumental. Participants may enter aesthetic settings under an expectation that nothing is at stake beyond the experience itself, and that framing plausibly alters the precision assigned to prediction errors encountered there, permitting model revision that a high-stakes setting would prompt the agent to defend against; the same property is what self-determination theory identifies as the autonomy of an activity undertaken for its own sake (Ryan & Deci, 2017), and what active inference describes as the pursuit of epistemic value under conditions that make exploration safe (Friston et al., 2015). This offers a mechanistic reading of why the same information can be assimilated in one frame and resisted in another.

The fourth is scale and cost. Artistic practices are ubiquitous across human cultures (Brown, 1991), which suggests that arts-based interventions may have relevance at population scale (Sacco, 2026a). Ubiquity alone, however, does not establish that specific interventions are uniformly accessible, scalable, or inexpensive to deliver. Within P3, efficiency must therefore be evaluated by relating the magnitude and durability of change to the resources required by matched interventions.

We do not claim that these candidate features are unique to the arts. Some, notably the third, are shared with play and with certain therapeutic formats. The hypothesis is that cultural forms combine them more consistently than the alternatives do, and it can be tested against the secondary comparator of P3, an affectively engaging but non-cultural format matched on arousal and social contact, and through dismantling designs that compare formats retaining or removing specific features on model-level change, persistence, transfer, and implementation cost.

## 5. Emotion, Metacognition, and Self-Regulated Learning

### 5.1. Metacognitive Monitoring as Partly Affective

The framework bears directly on metacognition, the monitoring and control of one’s own cognitive processes (Flavell, 1979; Nelson & Narens, 1990). Classical models describe a meta-level that monitors the object level and returns control signals to it. The integration account adds that the monitoring signals are, to a substantial part, affective. People judge whether they know an answer, whether a problem is tractable, or whether a conclusion is sound not by reading out the state of their cognitive machinery but by relying on cues experienced as feelings: fluency, familiarity, the feeling of knowing, and the feeling of rightness (Ackerman & Thompson, 2017; Koriat, 2000; Thompson et al., 2011). These metacognitive feelings are fast, low-effort appraisals that guide the allocation of cognitive resources. In the terms of Section 2.3, they can be understood as low-level estimates of the state of one’s own processing, and such estimates are felt rather than reasoned. A caveat is needed at once: the best-characterized cues, such as processing fluency and familiarity, are properties of information processing. Their specifically interoceptive origin remains conjectural and is supported so far only by limited evidence linking confidence to interoceptive signals (Critchley & Garfinkel, 2017).

The inferential character of these feelings is central. On cue-utilization accounts, metacognitive judgments are inferences drawn from cues such as the fluency with which information comes to mind, its familiarity, and the ease of processing, rather than direct readouts of memory strength or processing success (Koriat, 1997, 2000). Because the cues are heuristic, the resulting feelings can be systematically miscalibrated, as when fluency produced by superficial features inflates confidence. A useful distinction separates experience-based metacognition, driven by online affective and processing cues, from information-based metacognition, driven by explicit beliefs about one’s own cognition (Efklides, 2011). Both are needed, and both are shaped by affect: the first because the cues are themselves felt, the second because beliefs about one’s capacities carry emotional weight.

If metacognitive monitoring runs partly on affect, two consequences follow for intelligent performance. First, the quality of self-regulation depends on the calibration of these feelings, and calibration is learned through experience that carries the relevant affective signal rather than through instruction alone (Efklides, 2011). Second, interventions that dampen affect in the name of objectivity risk degrading the very signals on which accurate monitoring depends. Emotion regulation and metacognitive regulation appear, on this view, to be closely related achievements realized in overlapping systems: the prefrontal and cingulo-insular circuits implicated in the deliberate regulation of emotion (Etkin et al., 2015; Ochsner & Gross, 2005) overlap at a broad anatomical level with those implicated in metacognitive evaluation (Fernandez-Duque et al., 2000; Fleming & Dolan, 2012), though the specific subregions differ and co-location is only suggestive of a shared mechanism. This overlap is what the network account predicts, and it shifts the goal of intervention from suppressing affect toward cultivating well-calibrated affective monitoring.

To make such a goal empirically tractable, calibration must be measured, and the metacognition literature supplies the tools. A well-calibrated monitor is one whose confidence tracks accuracy, which has two separable aspects: metacognitive resolution or sensitivity, i.e., the degree to which higher confidence discriminates correct from incorrect responses, and metacognitive bias, the overall level of confidence independent of accuracy (Fleming & Lau, 2014; Nelson & Narens, 1990). Judgments of learning, feeling-of-knowing ratings, confidence-accuracy calibration curves, and bias-controlled measures such as meta-*d*′ provide established ways to quantify both, and to separate metacognitive efficiency from first-order performance, with which it is related but not identical (Fleming & Lau, 2014), much as confidence forms a factor of its own alongside the abilities measured by cognitive tests (Stankov & Crawford, 1997). Framing the claim in these terms sharpens it and exposes it to test. The prediction is that affective engagement improves resolution and calibration by supplying richer and more diagnostic feedback about the reliability of one’s own processing, and specifically not that it raises confidence, which would if anything risk overconfidence. That prediction is measurable, and its failure would provide disconfirming evidence.

### 5.2. Motivation, Achievement Emotions, and Self-Regulated Learning

Three motivational literatures connect the account to self-regulated learning, namely self-determination theory, achievement goal theory, and the models of self-regulated learning themselves, and each treats affect as a regulator of persistence, goal commitment, and the allocation of effort. Self-determination theory holds that regulation is sustained where the needs for autonomy, competence, and relatedness are satisfied, and that regulation originating outside the person becomes durable to the extent that it is internalized along a continuum running from external contingency through introjection and identification to integrated regulation (Ryan & Deci, 2017). Cultural and artistic practices are well placed to satisfy the three needs at once, supporting autonomy through the voluntary and self-expressive character of participation, competence through graded mastery, and relatedness through shared performance and reception. The distinction between autonomous and controlled regulation is itself graded, a continuum of the kind that an account built on configurations of shared machinery predicts, and it implies no partition into two kinds of process.

In achievement goal theory, the second of these literatures, mastery goals orient the learner toward the development of competence, whereas performance goals orient attention to comparative evaluation (Dweck & Leggett, 1988), and the motivational climate of a setting can promote either orientation, with mastery climates encouraging persistence, adaptive responses to error, and deeper engagement with the task (Urdan & Kaplan, 2020). The contrast is sharpened by the distinction between an empowering climate, which combines task-involving, autonomy-supportive, and socially supportive features, and a disempowering climate, which combines ego-involving and controlling ones (Duda, 2013; Milton et al., 2025). The construct converges, from a different literature, on the error framing of Section 4.3, since what a mastery climate does to the meaning of an error is what error-management training does to the meaning of a failed attempt (Keith & Frese, 2008), and the present framework proposes a common mechanism for both, in which the framing sets the precision assigned to the resulting prediction error and thereby determines whether it is used for revision or defended against. Because the same cultural activity can be organized either way, the regulatory benefit of cultural participation should depend on the climate in which it is delivered, which is a moderator that arts-based intervention studies rarely measure, although they could. These literatures also bear on the taxonomy of Section 3.3: a Type A challenge calls for no internalization, which is why a default suffices; a Type B challenge attenuates when environmental support substitutes for regulation without internalizing it, which is consistent with the finding that nudges work only toward options that people already want (de Ridder et al., 2022); and Type C change is close to what self-determination theory calls integrated regulation. The prediction that follows is specific: interventions that produce durable change on Type B and Type C challenges should show gains in need satisfaction and movement along the internalization continuum, and those gains should mediate the persistence of the effect once the intervention ends.

Models of self-regulated learning, the third of these literatures, provide the most developed applied setting for these ideas. Self-regulated learning is generally described as a cyclical coordination of forethought, performance, and reflection, in which learners set goals, monitor progress, and adjust strategy (Panadero, 2017; Winne & Hadwin, 1998; Zimmerman, 2002). Prominent models place monitoring and control at the center of the cycle (Winne & Hadwin, 1998). They also treat affect and motivation as regulated components in their own right, which condition the selection of strategies, the interpretation of monitoring, and the persistence of effort (Efklides, 2011; Pintrich, 2000; Zheng et al., 2023). A model of self-regulation that treats affect as noise to be minimized therefore misdescribes the process it seeks to support.

The most direct precedent for this position is the control-value theory of achievement emotions, on which emotions experienced in achievement settings arise from appraisals of control and value and in turn shape attention, strategy use, motivation, and self-regulation, with reciprocal effects over time (Pekrun, 2006, 2024; Pekrun et al., 2002). Control-value theory conceptualizes emotion as a determinant of learning outcomes rather than a byproduct of them, and it supplies the appraisal-level vocabulary that our account requires at the model level. A further finding is relevant to this argument: ability-based emotional intelligence shows a modest but reliable meta-analytic association with academic achievement (*ρ* = 0.24), which survives adjustment for intelligence and the Big Five and adds 1.7 percent of incremental variance, indicating that competence in the perception, understanding, and regulation of emotion contributes to intelligent performance in its own right, if modestly (MacCann et al., 2020; Mayer et al., 2008). The theoretical account is compatible with these findings and offers a mechanistic interpretation of them, on which the regulation of affect may be efficacious because it influences the accessibility of the states in which ability can be expressed.

### 5.3. Evidence from Affect- and Arts-Based Interventions

The intervention evidence in education is more heterogeneous than the theoretical case, and it must be read accordingly. School-based social-emotional learning programs lie outside the arts, though they share the emphasis on affective and social engagement. A contemporary meta-analysis encompassing 424 studies, 575,361 students, and 53 countries found significant improvements in social-emotional skills, attitudes, behavior, school functioning, and academic achievement relative to control conditions, although effects varied across interventions and contexts (Cipriano et al., 2023). A separate meta-analysis of 82 interventions involving 97,406 students found that benefits remained detectable between 6 months and 18 years after intervention (Taylor et al., 2017). We read this as indirect evidence that engaging affect and social relation is compatible with academic learning and can support it, which falls short of a demonstration of superiority over informational interventions, since that would require a direct comparison. Learning and brain development depend on social-emotional experience rather than on cognition operating in isolation (Immordino-Yang & Damasio, 2007; Immordino-Yang et al., 2019).

Arts-based interventions add a second line of evidence with complementary strengths and weaknesses. In two longitudinal quasi-experimental studies, elementary-school children and high-school students receiving a year of either acting training or training in the visual arts or music were assessed before and after. Acting students showed gains in self-reported empathy in both studies, whereas improvement on a naturalistic theory-of-mind measure was observed only among the adolescents (Goldstein & Winner, 2012). A preregistered randomized controlled trial assigned 235 Kenyan high-school students aged 13–19 years, 53.19% of whom were female, to one week of the Pre-Texts literature and arts-based protocol or an equal-duration study-skills control. Relative to the control condition, Pre-Texts produced greater reductions in self-reported depression and anxiety symptoms from baseline to one-month follow-up (Osborn et al., 2023). That the comparator was an active study-skills condition of equal duration, and not a waiting list, brings the trial closer to the comparison required to evaluate P3 than most of the available evidence, although the content of the two conditions differed and the outcomes were symptom measures, not the regulatory and metacognitive ones that we predict. At population scale, people who work in cultural and creative sectors and occupations show higher literacy, numeracy, and problem-solving skills than other workers in cross-national survey data (Sacco et al., 2025). The association is observational and cannot by itself separate an effect of the working environment from selection into it.

Outside education, the arts-and-health literature adds evidence of durability that is broader but weaker in design. In a systematic review and meta-analysis of prospective studies, benefit from music therapy for people with serious mental disorders increased with the number of sessions delivered (Gold et al., 2009); receptive arts engagement was associated with lower mortality over fourteen years of follow-up in a cohort of 6,710 adults aged fifty and over (Fancourt & Steptoe, 2019); and a major scoping review documents a wide range of outcomes while noting that the field is still consolidating common outcome measures and guarding against publication bias (Fancourt & Finn, 2019). A recent review describes fifty candidate causal mechanisms through which arts engagement acts on mental health, concludes that combinations of interconnected mechanisms are likely to explain its effects, and proposes that everyday patterns of engagement form an arts exposome that activates them (Fancourt et al., 2026), which shows how far the field remains from attributing an effect to any single mechanism, including those that we emphasize. Much of this evidence is observational, so its value here is convergent: several independent lines associate affectively and socially engaging practices with durable change, without bearing on the comparison that P3 requires.

The educational literature does not yet contain the direct comparison required to evaluate P3. To our knowledge, no study has compared a culturally mediated intervention against a content-matched control on metacognitive resolution, on the maintenance of self-regulated learning under difficulty, or on transfer, with follow-up long enough to test durability. The literature therefore constrains our account without confirming it; Section 5.5 states the corresponding predictions, and Section 7.3 outlines a test.

### 5.4. Metacognition as Hierarchical Inference

The predictive framework of Section 2.3 and the metacognitive account developed here can be brought together. If first-order perception and action are cast as inference over the causes of sensory and interoceptive signals, then metacognition can be cast as inference at a higher level about the reliability of that first-order inference, that is, about its precision (Fleming & Dolan, 2012; Friston, 2010). A feeling of confidence, on this reading, is an estimate of the expected accuracy of a judgment, and a feeling of difficulty an estimate that first-order processing is proceeding with low precision. The construal has two desirable qualities: it explains why monitoring is experienced affectively rather than as a dispassionate report, because precision estimates are plausibly bound up with the interoceptive and allostatic signals through which the brain tracks its own regulatory state, though this specific grounding remains conjectural. It also clarifies why the calibration of monitoring can be trained, since adjusting the mapping from cues to confidence is itself a form of learning that depends on affectively marked feedback. Metacognition, on this view, is also distributed beyond the individual, and calibrated by social and cultural feedback against shared standards (Shea et al., 2014). The practical implication is that cultivating accurate metacognition is less a matter of adding a supervisory layer above cognition than of tuning the affective signals through which the system already estimates its own reliability, and structured, low-threat cultural practices are candidate means of achieving this.

### 5.5. Predictions for Metacognition, Learning, and Intelligent Performance

The claims of this section are, at present, predictions rather than findings. Three are implied by the framework and are in principle testable with existing methods. First, affectively and socially scaffolded practice should improve metacognitive resolution and calibration on transfer tasks, measured as in Section 5.1, rather than merely raise confidence or task-specific performance. Second, such practice should improve the maintenance of self-regulated learning under difficulty, in persistence, strategy adjustment, and error tolerance, more than content-matched controls, with effects that outlast the intervention. Third, the benefit should be systematic rather than uniform across individuals, patterning with baseline metacognitive efficiency, trait aesthetic sensitivity, and the regulatory demands of the task.

The connection to intelligence runs through the second and third predictions. Intelligent performance in real settings depends on the regulated deployment of cognitive ability as well as on its possession: on the protection of attention and working memory under load, the accurate monitoring that decides when to persist and when to switch, and the affective tolerance that keeps difficult problems open, which together decide how far typical performance falls short of maximal performance (Sackett et al., 1988). The regulation of attention is among the most robust correlates of fluid reasoning (Engle, 2002); metacognitive efficiency is separable from first-order accuracy (Fleming & Lau, 2014); and the tendency to reason well is only weakly predicted by cognitive ability itself (Stanovich & West, 2008), which leaves substantial room for regulatory and affective determinants of performance. Our framework locates the affective and cultural scaffolding of these regulatory capacities and predicts that interventions targeting them should show their clearest benefits where the expression of ability is bottlenecked by regulation rather than by capacity itself. The transfer that these predictions concern is near transfer of a regulatory and metacognitive skill to untrained tasks and settings that make similar demands, and the account predicts no gain in general cognitive ability, which well-controlled meta-analyses of music training do not find (Sala & Gobet, 2017, 2020). This is the boundary stated in Section 1.1: the paper concerns the regulated use of intelligence, and its claims about intelligent performance should be read at that level.

## 6. Informational and Aesthetic Delivery: An Illustrative Case

The case examined in this section concerns intergroup prosociality, outside the domain of learning and metacognition, and it serves to show why the comparison that P3 requires has to be run, since the same target belief was addressed in it both by brief textual delivery and by embodied, aesthetic delivery. Intergroup empathy across an active conflict line is a Type C challenge in a demanding form, because the target is a generative model of the outgroup that is defended by identity, sustained by community, and updated in ordinary life only through infrequent and costly contact. The case bears on learning by analogy, and it provides no evidence for the predictions of Section 5.5.

A series of laboratory and field studies targeted lay theories of empathy, the implicit belief that one’s own empathic capacity is either limited or expandable (Hasson et al., 2022). Such beliefs belong to the family of implicit theories that shape motivation and self-regulation (Dweck & Leggett, 1988), the family to which implicit theories of intelligence also belong, and because they operate across situations, changing them could produce domain-general shifts in emotional availability. Across six studies with 2118 participants in Israel and the United States, belief in unlimited empathy was associated with greater empathy toward outgroup members in a sample of 1308 Israeli adolescents, with modest correlations (roughly *r* = 0.16 to 0.25), and brief texts presenting empathy as limited or expandable shifted the targeted belief and raised reported empathy toward refugees in the unlimited condition (*d* = 0.42 in the online study). In one field implementation, the manipulation was delivered by trained actors within a theatrical set, with participants randomly assigned to conditions; in another, at a festival in Jerusalem, 176 Israeli Jewish participants encountered Jewish and Arab performers recounting difficult personal stories and then chose whether to end the encounter with no contact, a handshake, or an embrace. Control participants showed the expected ingroup bias, whereas in the unlimited condition that bias was attenuated and physical contact with the outgroup performer increased. Participants self-selected into the events, outcomes were measured close to the intervention, and the report characterizes the behavioral differences categorically, without standardized effect sizes for the choice outcome.

Five preregistered, high-powered online studies (*n* = 4776) have since found that the brief textual manipulation does not increase empathy or prosocial behavior toward outgroup members relative to control, including a study that used the original stimuli and one that reinstated the original interventions alongside the original control condition, although the induced belief shifted as intended in most studies (Tagesson et al., 2025). The limited condition often reduced empathy, so that withholding proved easier to induce than extending, and in the closest replication the attenuated intergroup bias in the unlimited condition reappeared, but it reflected lower empathy toward ingroup targets than in the control conditions, with empathy toward outgroup targets unchanged across conditions, which points to a mechanism different from the one originally proposed. What failed to replicate is the brief, textual, appraisal-level delivery, which the present account regards as the weakest route to model-level change. The embodied and aesthetic field implementations have not been tested independently, so the replication record is silent on the route that P3 specifies, and the non-replication of the textual arm cannot be read as evidence for the aesthetic arm, since a single unreplicated field study is weak evidence whatever happens to its laboratory companion.

The case therefore makes the matched comparison a necessary test, and it shows what the comparison has to hold fixed. On the account developed here, embodiment, collective synchrony, and aesthetic framing are the features through which a model-level change should hold and generalize where a text alone would not, so the required design delivers the same target belief through informational and through performance-based formats, matched in content and dose, and measures persistence and transfer at follow-up. Section 7.3 outlines a comparison of this kind for the domain of learning.

## 7. Discussion

### 7.1. Evidential Standing, Scope Conditions, and Disconfirmation

Table 3 assigns a grade to each link on which the account depends, using the five grades defined in Section 1.1. The pattern it shows is the one that the propositions state: the integration of emotion and cognition rests on direct and convergent evidence, the mechanisms through which cultural practices might act rest on indirect evidence or on inference, and the links from those mechanisms to reduced regulatory effort, model revision, and durable change are hypotheses, with the extension to formal control energy a conjecture.

The scope of P3 follows from this standing. It concerns Type B and Type C challenges, for which a culturally mediated route is hypothesized to be more efficient, more durable, and more transferable than a content-matched route without affective, embodied, social, and aesthetic design features; for Type A challenges, choice architecture is typically the efficient instrument, subject to the revision that its own evidence base is currently undergoing (Section 3.3), and nothing in our argument suggests otherwise. The corresponding denials are equally part of the claim: no sufficiency, no uniform superiority of cultural interventions over alternatives, and no suggestion that cognitive and environmental determinants of behavior are dispensable. Response is also expected to vary systematically, since controllability varies across individuals with white-matter architecture (Gu et al., 2015), aesthetic appeal is represented in default-mode regions in a markedly individual-specific way (Vessel et al., 2012, 2019), personality has been investigated as a moderator of the well-being returns to arts participation in longitudinal panel data (Weziak-Bialowolska et al., 2019), and ability-based emotional intelligence carries a small but reliable association with achievement (MacCann et al., 2020). Whether those who begin with higher barriers to adaptive states benefit most from scaffolding or least, and whether receptive engagement differs in effect from active artistic production, which involves deeper motor simulation and personal meaning-making, are open questions, and studies should model heterogeneity of response instead of averaging over it.

The strongest challenge to this position comes from interventions that are comparatively cognitive yet produce durable change. Implementation intentions, brief plans that link situational cues to intended responses, have reliable effects on goal attainment across domains (Gollwitzer, 1999; Gollwitzer & Sheeran, 2006). Cognitive-behavioral therapy produces reductions in symptoms that persist after treatment ends (Hofmann et al., 2012; Hollon et al., 2005). Training in self-regulated learning improves both self-regulatory strategy use and academic performance (Theobald, 2021). These cases constrain the hypothesis, and their bearing on it depends on their design profile, since affective engagement is defined by design, independently of outcome (Section 1.1). Cognitive-behavioral therapy works substantially through emotional processing and exposure, so it is affectively engaging by design without the aesthetic and symbolic features of cultural forms; its durability bears on the secondary comparison of P3, whether cultural forms add something to affective engagement, and leaves the primary comparison untouched. Implementation intentions and most boosts are strategic and informational, and they are the cases that press hardest on the primary comparison. Self-regulated-learning training varies in profile, since some programs add motivational and affective components to strategy instruction, and each program enters the comparison according to the design features that it carries.

Two claims should therefore be kept apart. The first is about level: behavioral change that persists and transfers tends to require reorganizing the appraisals and predictive models from which behavior flows, an inference consistent with the evidence reviewed in Section 3 and Section 5, and both cognitive-strategic and affective-cultural interventions can operate at that level. The second, which is the hypothesis specific to this article, is about route: for Type B and especially Type C challenges, the culturally mediated route to model-level change is more efficient, more durable, and more transferable than the content-matched alternative, and in some settings it may be the only practically available one.

The route hypothesis would fail if interventions built without emotional, interoceptive, embodied, social, or aesthetic components, including strategic and metacognitive interventions as well as informational or incentive-based ones, were shown to match or exceed culturally mediated ones in producing self-regulatory change that persists without environmental support and generalizes to untrained contexts, for challenges of Type B and Type C. Persistence alone would not settle the comparison, since habit can sustain a behavior within a stable context (Section 3.3), and the transfer criterion is what separates a change in the beliefs that govern behavior from cue-bound automaticity. The narrower claim about what is specific to cultural forms would fail if affectively engaging but non-cultural formats, matched on arousal and social contact, equaled the culturally mediated ones on the same outcomes.

### 7.2. From Constructs to Measures

The propositions of Section 1.2 are stated in terms of high-level constructs, and their testability depends on whether each construct can be measured with instruments already in use. Table 4 lists the principal constructs, the working definition that we adopt, the measures that we would use, and the direction of the effect that the account predicts. Three features of the table carry the argument. Affective engagement is defined by design, independently of outcome, as Section 1.1 specifies, and is verified by a manipulation check rather than inferred from success. Cost is separated into the subjective regulatory effort that current methods can measure and the formal control energy that they cannot yet measure for the regulatory states at issue, so that a study can test the propositions without relying on the neural conjecture. And the outcomes that distinguish the account from its rivals are calibration, durability, and transfer rather than immediate performance, since a route that merely raised performance at the point of delivery would be consistent with almost any framework. For design purposes, the entries also fix what the primary comparator of P3 must hold constant, namely content, contact time, delivery intensity, group format, and facilitator contact, leaving the affective, embodied, social, and aesthetic design features as the manipulated difference.

**Table 4:** Operationalization of the principal constructs, with the direction of effect predicted by the propositions of Section 1.2.

Construct	Working Definition	Measurement	Predicted Direction
Affective engagement	An intervention built to recruit emotional, interoceptive, embodied, social, or aesthetic processes, fixed before any outcome is observed	Design coding against pre-specified criteria; manipulation checks on arousal, felt involvement, and interpersonal synchrony	Present by construction in the treatment arm and absent in the matched comparator
Regulatory effort	The difficulty of reaching and holding a state in which demanding work can be undertaken	Effort and workload ratings, persistence under load, pupillometry, cardiac indices of exerted control, the temporal profile of performance across sustained tasks	Lower in the affectively scaffolded arm at matched task difficulty
Control energy	The input required to steer the modeled system between states on the structural connectome	Network control theory metrics derived from diffusion and functional imaging (Parkes et al., 2024)	Conjectural; no directional prediction is committed at this level
Metacognitive resolution	The degree to which confidence discriminates correct from incorrect responses	meta-d′, area under the type 2 ROC, confidence-accuracy calibration curves (Fleming & Lau, 2014)	Higher after affectively scaffolded practice, and on transfer tasks rather than only on trained ones
Metacognitive bias	The overall level of confidence independent of accuracy	Mean confidence and over- or underconfidence indices (Fleming & Lau, 2014)	No predicted increase; a rise in confidence without a gain in resolution counts against the account
Maintenance of self-regulated learning	The regeneration of monitoring, strategy use, and effort allocation across occasions of study	Learning-trace and log data, strategy-shift counts, persistence after failure, established self-report instruments	Better maintained after the intervention ends than in matched comparators
Model-level change	Revision of the beliefs, appraisals, or predictive models governing a class of behaviors	Implicit-theory and epistemic-belief measures, appraisal measures, generalization to untrained situations	Larger and more general than in informationally matched comparators
Need satisfaction and internalization	Satisfaction of autonomy, competence, and relatedness, and position on the internalization continuum	Basic psychological need satisfaction scales and perceived locus of causality (Ryan & Deci, 2017)	Higher in the affectively scaffolded arm, and mediating the durability of the effect
Motivational climate	Task-involving and autonomy-supportive as against ego-involving and controlling delivery of the same activity	Climate questionnaires and observational coding of delivery (Duda, 2013)	Moderates the effect of an otherwise identical activity
Durability	Persistence of change after the withdrawal of support	Follow-up at intervals long enough to observe decay, with no further contact between measurements	Slower decay than in matched comparators
Transfer	Persistence of change in tasks and contexts in which the behavior was not practiced	Untrained tasks and settings assessed at each follow-up	Larger than in content-matched comparators; persistence without transfer is consistent with habit and does not discriminate between routes
Efficiency	Change obtained per unit of resource	Effect size per participant-hour and per unit of delivery cost, reported alongside magnitude	Favors the cultural route for Type B and Type C challenges

### 7.3. A Proposed Test of the Route Hypothesis

The comparison that P3 requires can be specified for an educational setting with instruments already in use, and Table 5 outlines a design that could be preregistered. The setting is a course with high self-regulatory demand, such as the first year of a university degree, in which classes are randomized as clusters to three arms: a culturally delivered program that teaches self-regulated-learning strategies and an informative framing of error through ensemble, theatrical, or narrative practice; a content-matched strategic program that teaches the same strategies and framing through instruction and guided practice, with the same contact time, intensity, group format, and facilitators; and an affectively engaging but non-cultural program, such as cooperative games without aesthetic form, matched on arousal, social contact, and physical exertion, which tests what is specific to cultural forms. The two primary outcomes are chosen to discriminate between the account and its rivals. Metacognitive efficiency on tasks that were not trained separates a gain in calibration from a rise in confidence, and the maintenance of self-regulated learning after the program ends, measured from learning-trace data, separates durable change from change sustained by the program itself; persistence is assessed together with transfer, because persistence alone could reflect habits formed within the program’s context. Metacognitive efficiency is estimated hierarchically (Fleming, 2017) from enough trials per participant for acceptable reliability, with first-order performance modeled alongside it, since no current measure is independent of first-order performance and reliability falls at the trial numbers common in the field (Guggenmos, 2021). General cognitive ability is measured at baseline and at the last follow-up as a discriminant outcome, for which no difference between arms is predicted, so that a gain in calibration or maintenance cannot be read as a gain in capacity.

Manipulation checks verify that the culturally delivered arm engages affect and that the strategic arm does not, and that the non-cultural arm matches the culturally delivered one on arousal and social contact while lacking its aesthetic and symbolic features; fidelity checks verify that content is matched, and the moderators named in Section 1.2 are measured at baseline. The primary contrast sets the culturally delivered arm against the strategic arm, with the two primary outcomes tested under a correction for multiplicity and the non-cultural arm entering a secondary contrast, and outcomes are assessed blind to arm under a prespecified treatment of attrition. The disconfirmation condition of Section 7.1 then takes a form that can be preregistered: P3 fails if the strategic arm matches or exceeds the culturally delivered arm on both primary outcomes at the six- and twelve-month follow-ups, and a rise in confidence without a gain in resolution counts against the account whatever happens to performance. The scale of such a study is substantial: for the primary comparison, a smallest effect of interest of *d* = 0.25 at follow-up, and a two-sided α of 0.025 for each primary outcome, power of 0.80 requires 306 students per arm under individual randomization, and with twenty-five students per class and an intraclass correlation of 0.10 the design effect of 3.4 raises this to about 1,040 students, or 42 classes, per arm, before any gain from adjustment for baseline covariates. A quicker test is available in the domain of intelligence itself, since the growth-mindset module of the national experiment (Yeager et al., 2019), delivered as designed or as the same content in narrative or theatrical form of equal duration, would test P3 for a Type C target with an outcome infrastructure, grades and measured belief change, already in place, and the test would count against P3 if the narrative or theatrical version failed to outperform the module on grades or on measured belief change at follow-up.

### 7.4. Implications for Intervention and Educational Design

Several provisional design principles follow from the theoretical account, and their evidential strength is limited by the evidence for the mechanisms on which they rest. Interventions should make target states easier to reach and hold instead of relying on sustained effortful control; synchronization, arousal modulation, and external scaffolding are, on our account, the levers that cultural practices characteristically use to this end. They should frame error as informative, preserving the prediction-error signal that drives learning while removing its threat. They should engage embodied and interoceptive processes, since the affective signals that guide monitoring are bodily grounded, which argues for participatory rather than purely receptive designs where feasible.

In education specifically, these principles align the support of self-regulated learning with social-emotional engagement rather than treating them as separate agendas (Durlak et al., 2011; Immordino-Yang & Damasio, 2007; Pekrun, 2006). In classrooms, this means embedding the regulation of learning within affectively and socially meaningful activity: tasks that carry narrative or personal stakes, formative feedback framed to preserve the informational value of error without its threat, and collaborative formats that recruit interpersonal synchrony. The design implication that follows most directly from Section 4.4 is that the cultural material should be kept structurally intact rather than reduced to whichever component seems mechanistically active, since the features that we have identified as distinctive, and in particular the combination of symbolic content with a voluntary and low-stakes frame, are properties of the practice as a whole.

### 7.5. Limitations and Future Directions

The proposal is programmatic, and its limitations begin with the fact that affective engagement is not uniformly beneficial, since arousal beyond the productive range degrades performance (Arent & Landers, 2003) and anxiety in particular impairs the goal-directed attentional control on which monitoring depends (Eysenck et al., 2007). The proposed link between cultural engagement and network controllability remains conjectural; the priority is direct evidence connecting defined cultural interventions to controllability metrics, distinguishing acute functional change from structural plasticity, and tracing the path from practice through neural change to behavior. Comparative effect sizes between affective and choice-architecture interventions cannot at present be stated with confidence, because outcomes, time horizons, and cost conventions differ across the relevant literatures, and because the two families of interventions have rarely been applied to the same challenge; establishing common metrics and running head-to-head comparisons of the kind outlined in Section 7.3 is a precondition for the comparative claims that the field will want to make. A further obstacle is that the choice-architecture side of the comparison rests on meta-analyses of mostly low methodological quality, whose bias-adjusted estimates range from small to null (Hu et al., 2025), so that a comparison conducted now would evaluate P3 against an unstable benchmark. The field evidence we have discussed involves self-selected participants and immediate outcomes, so longitudinal designs with more representative samples and active controls are needed to establish durability and generality. The replication record reviewed in Section 6 sharpens this requirement, since the laboratory arm of the illustrative case did not replicate and its aesthetic and embodied arm has not been tested independently, so that the case motivates the comparison without supporting the account.

The lifespan dimension is a further gap. If the transition landscape differs by age, as the cross-sectional developmental evidence discussed in Section 4.1 suggests it may, a given intervention will meet different conditions at different ages, and whether periods of heightened plasticity offer special opportunity is a conjecture worth testing. Metacognitive skills likewise develop across childhood and adolescence and continue to be refined by education (Efklides, 2011; Flavell, 1979), and we have no direct evidence on how affective scaffolding of the kind described here interacts with these trajectories. Individual differences in response call for models that match people to modalities (Section 7.1).

Cultural specificity raises a further limitation, since interventions adapt their surface features, such as aesthetic forms, narrative content, and movement vocabularies, to the sensibilities of their participants, while the core mechanisms that they recruit, namely belief revision, embodied simulation, and synchrony-based interpersonal coupling, may operate more widely because the capacities involved recur across human societies (Brown, 1991). This distinction must be handled with care, because the constructed-emotion account that we endorse holds that emotion concepts are themselves culturally shaped (Barrett, 2017), and cultural differences in how emotion is read from context are well documented (Masuda et al., 2008). The two claims are compatible if construction operates at the level of concepts and experience while the underlying predictive and interoceptive architecture is shared (Barrett & Satpute, 2013), but whether a belief such as the unlimited-empathy belief of Section 6 carries equivalent meaning across cultures with differing construals of selfhood is an empirical question, and cross-cultural work attentive to indigenous concepts of emotion and selfhood is needed to settle it.

Finally, affective and cultural practices are not intrinsically benign: poorly facilitated group processes can entrench bias, powerful aesthetic experiences can retraumatize, and the mechanisms that support prosociality can be turned to manipulation, as the history of propaganda art attests. Boundary conditions and safeguards belong firmly within the research program and its implementation.

## 8. Conclusions

Intelligent performance depends on the coordination of cognition, self-regulation, metacognition, and emotion, and the evidence reviewed here indicates that these are aspects of integrated network dynamics rather than separable faculties. The account we have developed concerns the regulated deployment of ability, on which the distance between maximal and typical performance depends, and it leaves the structure of ability itself outside its scope. Read in that light, the treatment of emotion as a bias to be overridden and the intervention strategies built upon it address the part of the problem in which a single decision settles behavior. For behaviors that must be regenerated over time, and for the beliefs from which behavior flows, the states that need to be reached are, on our account, affectively constituted, which makes interventions that engage affect a promising route to durable change; whether they are the more efficient, durable, and transferable route is the hypothesis that now needs to be tested. Cultural and artistic practices, refined over the long history of human societies, are a candidate means of providing that engagement, since they can synchronize participants, hold arousal within productive bounds, reframe error as information, and support the revision of the predictive models through which people understand themselves and others.

We have advanced a graded hypothesis about the relative merits of two routes to change, which allows for durable effects from some comparatively cognitive interventions and treats neither affect as sufficient nor culture as uniquely privileged. Its content is testable in the near term without waiting on the neural conjecture: the predictions of Section 5.5 concern metacognitive resolution, the maintenance of self-regulated learning under difficulty, and the patterning of benefit across individuals, and each can be assessed with instruments already in use. The disconfirmation condition set out in Section 7.1 states what would count against the hypothesis, and the replication record reviewed in Section 6 is a reminder that the case which motivated it is not itself settled. Running the comparison outlined in Section 7.3, against a content-matched control and with follow-up long enough to speak to durability and transfer, is the natural next step.

## Figures and Tables

**Table 1:** Three classes of behavioral challenge, classified by regulatory structure, with predicted intervention fit.

Class	Defining Feature	Exemplars in Behavior Change	Exemplars in Learning and Metacognition	Predicted Intervention Fit
Type A	The target behavior, once selected, is self-maintaining and requires no further regulation	Organ-donor registration, retirement-savings enrollment, default placement of options	Course or track enrollment, opt-in to a study-support service	Choice architecture predicted to be effective and efficient
Type B	The behavior must be produced repeatedly against competing demands, so the regulatory demand recurs over time	Weight management, medication adherence, exercise maintenance, reduction of substance use	Sustained study practice, persistence under difficulty, strategy adjustment across a course	Choice architecture predicted to shift behavior with effects that attenuate once environmental support is withdrawn, except where engineering removes the regulatory demand or habit makes the behavior self-maintaining within a stable context
Type C	The target is a generative model, so that change requires reorganizing the beliefs, appraisals, or predictive models from which a range of behaviors flows	Intergroup empathy, prejudice reduction, shifts in self-relevant values	Implicit theories of one’s own ability, epistemic beliefs, the calibration of metacognitive feelings	Choice architecture predicted not to reach the model level by itself; culturally mediated routes predicted to be more efficient, more durable, and more transferable than content-matched ones

**Table 2:** The classification rule applied. Q1: does the behavior persist without further regulatory input once selected? Q2: must it be produced again on each occasion against competing demands? Q3: is the target a belief, appraisal, or model governing a family of behaviors? A dash marks a question that the rule does not ask.

Target Behavior and Declared Outcome	Q1	Q2	Q3	Class	Comment
Organ-donor registration (registration)	Yes	—	No	A	Default placement is sufficient
Retirement saving (enrollment)	Yes	—	No	A	One policy, first declared outcome
Retirement saving (adequacy over a career)	No	Yes	No	B	The same policy under a second declared outcome, which is why the declaration matters
Medication adherence (daily oral regimen)	No	Yes	No	B	The regulatory demand recurs on every occasion
Medication adherence (long-acting formulation)	Yes	—	No	A	Engineering removes the demand instead of building regulation
Sustained study practice across a course	No	Yes	No	B	Monitoring and effort allocation are regenerated each session
Implicit theory of one’s own ability	No	No	Yes	C	A model that governs persistence across many tasks
Intergroup empathy across an active conflict line	No	No	Yes	C	The case of Section 6, in a demanding form
Calibration of metacognitive feelings (accuracy of monitoring during a course)	No	Yes	Yes	Mixed, B dominant	Monitoring is regenerated in each session; the cue-to-confidence mapping is recorded as the subordinate component
Calibration of metacognitive feelings (resolution on untrained transfer tasks)	No	Yes	Yes	Mixed, C dominant	The same challenge under a second declared outcome, carried by a cue-to-confidence mapping that has to be relearned
Dietary change for weight management	No	Yes	Yes	Mixed, B dominant	Assigned to B because maintenance dominates variance in the declared outcome; the model component is recorded as subordinate

**Table 3:** The evidential standing of the links on which the account depends, graded as defined in Section 1.1.

Link	Standing	Basis
Emotion and cognition are realized in overlapping, reciprocally connected networks (P1)	Direct evidence	Convergent neuroanatomical, neuroimaging, and connectivity findings (Okon-Singer et al., 2015; Pessoa, 2017)
Processing features dissociate across tasks, as configurations of shared machinery would produce	Direct evidence for the dissociation; inference for its explanation	Melnikoff and Bargh (2018); its bearing on the defining features is contested (Pennycook et al., 2018); Section 3.1
Choice-architecture effects are small and heterogeneous	Direct evidence	Second-order meta-analysis of 14 meta-analyses (Hu et al., 2025); the domain pattern is not significant
Effects of repeated interventions decay slowly once they stop, leaving a residue	Direct evidence in a single domain; inference beyond it	Home energy reports (Allcott & Rogers, 2014); untested in other domains
Change that persists and transfers requires reorganized appraisals and predictive models (P2)	Inference	Qualified by habit research (Wood & Rünger, 2016); Section 3.3
Collective music-making synchronizes brain activity, and aesthetic experience engages reward and default-mode circuitry	Direct evidence from small samples	Müller et al. (2013); Salimpoor et al. (2011); Vessel et al. (2012)
These responses reflect shared predictive states and an integration of reward and self-referential processing	Inference	Section 4.2; common sensory and motor input is an alternative explanation
Informative error framing improves adaptive transfer	Direct, meta-analytic evidence in training settings; inference for cultural settings	Keith and Frese (2008); Steele-Johnson and Kalinoski (2014)
Framing sets the precision assigned to prediction error, and so whether a model is revised	Hypothesis	Section 4.3 and Figure 3
Cultural engagement lowers the subjective effort of reaching and holding adaptive states (P4)	Hypothesis	Measurable with effort ratings, persistence, and physiological indices (Table 4); untested
Cultural engagement lowers the formal control energy of brain-state transitions (P4)	Conjecture	No study links a cultural intervention to controllability metrics (Section 4.2)
Affective engagement improves metacognitive resolution and calibration	Hypothesis	Section 5.5; untested
A culturally mediated route yields more efficient, durable, and transferable change than a content-matched one (P3)	Hypothesis	Indirect support from affective and arts-based programs with lasting or comparator-controlled effects (Osborn et al., 2023; Taylor et al., 2017); no direct comparison
Cultural forms add value beyond affectively engaging formats that are not cultural	Hypothesis	Section 4.4; untested
Aesthetic delivery of a belief change produces durable intergroup change	Direct evidence from one unreplicated field study; durability untested	Hasson et al. (2022); the textual arm did not replicate (Tagesson et al., 2025)

**Table 5:** Outline of a test of the route hypothesis (P3) in an educational setting, in a form that could be preregistered.

Element	Specification
Design	Cluster-randomized trial in which classes of a course with high self-regulatory demand are assigned to three arms
Arms	(1) Culturally delivered program teaching self-regulated learning strategies and informative error framing through ensemble, theatrical, or narrative practice; (2) content-matched strategic program; (3) affectively engaging non-cultural program, such as cooperative games without aesthetic form, matched on arousal, social contact, and physical exertion
Held constant	Content, contact time, delivery intensity, group format, facilitators, and expected benefit rated by participants before the program
Primary outcomes	Metacognitive efficiency (meta-d′/d′) on untrained tasks, estimated hierarchically from enough trials for acceptable reliability, with first-order performance modeled; maintenance of self-regulated learning in learning-trace data after the program ends
Secondary outcomes	Subjective regulatory effort under load; persistence after failure; implicit theories of ability; need satisfaction and internalization; achievement emotions; course grades as a typical performance outcome
Discriminant outcome	General cognitive ability at baseline and final follow-up, with no difference between arms predicted
Manipulation and fidelity checks	Arousal, felt involvement, interpersonal synchrony, and aesthetic engagement in each arm; coding of delivered content against the protocol
Moderators	Baseline metacognitive efficiency; aesthetic responsiveness (Schlotz et al., 2021); motivational climate (Duda, 2013)
Follow-up	End of program and 3, 6, and 12 months later, with no contact between measurements
Analysis	Primary contrast of arm 1 against arm 2 on both primary outcomes, corrected for multiplicity; arm 3 in a secondary contrast; multilevel models with classes as clusters; assessors blind to arm; prespecified treatment of attrition
Cost	Delivery cost per participant-hour, reported with the effect size obtained per unit of cost
Disconfirmation	Arm 2 matching or exceeding arm 1 on both primary outcomes at 6 and 12 months; a rise in confidence without a gain in resolution
Sample size	Smallest effect of interest at follow-up, with α divided between the two primary outcomes and the result inflated by the design effect of cluster randomization

## Data Availability

No new data were created or analyzed in this study. Data sharing is not applicable to this article.
